# The intersection of the HER2-low subtype with endocrine resistance: the role of interconnected signaling pathways

**DOI:** 10.3389/fonc.2024.1461190

**Published:** 2024-11-22

**Authors:** Gizem Yayli, Alexa Tokofsky, Utthara Nayar

**Affiliations:** ^1^ Department of Biochemistry and Molecular Biology, Bloomberg School of Public Health, Johns Hopkins University, Baltimore, MD, United States; ^2^ Oncology, Sidney Kimmel Comprehensive Cancer Center, School of Medicine, Johns Hopkins University, Baltimore, MD, United States

**Keywords:** endocrine resistance, combination therapy, HER2 mutations, ER-HER2 crosstalk, subtype switch

## Abstract

Since its introduction in the 1970s, endocrine therapy that targets the estrogen receptor alpha (ERα) signaling pathway has had tremendous success in the clinic in estrogen receptor positive (ER+) breast cancer. However, resistance to endocrine therapy eventually develops in virtually all patients with metastatic disease. Endocrine resistance is a primary unaddressed medical need for ER+ metastatic breast cancer patients. It has been shown that tumors become resistant through various mechanisms, converging on the acquisition of genetic alterations of ER, components of the MAP kinase pathway, or transcription factors (TFs). For instance, mutations in the human epidermal growth factor receptor-2 (HER2) lead to complete resistance to all current endocrine therapies including aromatase inhibitors, selective estrogen receptor modulators, and selective estrogen receptor degraders, as well as cross-resistance to CDK4/6 inhibitors (CDK4/6is). Emerging evidence points to an intriguing connection between endocrine-resistant tumors and the HER2-low subtype. Specifically, recent studies and our analysis of a publicly available breast cancer dataset both indicate that metastatic ER+ breast cancer with endocrine resistance conferred through acquired genetic alterations can often be classified as HER2-low rather than HER2-0/HER2-negative. Limited data suggest that acquired endocrine resistance can also be accompanied by a subtype switch. Therefore, we suggest that there is an underappreciated association between the HER2-low subtype and endocrine resistance. In this perspective piece, we explore the evidence linking the HER2-low subtype with the various pathways to endocrine resistance and suggest that there are signaling networks in HER2-low tumors that intersect endocrine resistance and can be effectively targeted.

## 1 Introduction

Human epidermal growth factor receptor-2 (HER2)-low breast cancer (BC) accounts for between 45% and 55% of all BC cases (1). By definition, they belong to luminal-like [estrogen receptor-positive (ER+)] and triple-negative BC (TNBC) subtypes with a lack of HER2 (*ERBB2*) amplification (1, 2). At least 80%–90% of HER2-low are ER+ [luminal A (LumA) or B (LumB)] tumors (3), as opposed to ~70% of HER2-0.

LumA or B ER+ BC is classically treated using ER (*ESR1*)-directed endocrine therapies—aromatase inhibitors (AIs) or the selective ER modulator (SERM) tamoxifen in the first-line setting, and the selective ER degrader (SERD) fulvestrant or the combination of endocrine with CDK4/6 inhibitor (CDK4/6i) palbociclib or ribociclib, which were tested in the PALOMA (4) and MONALEESA (5) trials, or CDK4/6i abemaciclib monotherapy (MONARCH (6) study), in the first-line and endocrine refractory second-line settings (7, 8). Endocrine resistance to ER inhibitors (ERis) is ubiquitous and a primary contributor to BC-associated mortality.

Endocrine resistance has been studied for decades using cell line models cultured to resistance—regardless of the specific cell line or the endocrine treatment, nearly all such models upregulate HER2 and/or HER2 binding partners (9–11). LumB tumors, which are characterized by increased HER2 expression and pathway activity signature compared to LumA, have worse outcomes on endocrine therapy (12). Thus, HER2 activity has a strong inverse correlation with endocrine sensitivity.

In recent years, large-scale sequencing studies have begun to shed light on the mechanisms of endocrine resistance that arise in the clinical setting (13, 14). Overall, these appear to converge on three major classes: 1) alterations in the target ER through mutations or novel oncofusions (15, 16), 2) transcription factor (TF) alterations, and 3) MAP kinase pathway-associated alterations, including upstream receptor tyrosine kinases (RTKs) and downstream components of the MAP kinase or PI3K/AKT/mTOR signaling cascades (14). Up to 60% of endocrine-resistant tumors do not harbor a genetic alteration in any of these classes, and this remains an area of active study.

Of the known mechanisms of clinical endocrine resistance, ER (*ESR1*) mutations (ERmuts) are the most common, accounting for 18%–50% of resistant tumors depending on patient cohort characteristics (higher prevalence in AI-treated populations) (17–20). ERmuts have been extensively studied (21) and can be clinically targeted with novel oral SERDs, the first of which elacestrant (22) was Food and Drug Administration (FDA) approved for ERmut tumors in 2023. At the same time, the other specific alterations acquired in resistant tumors are incredibly diverse and, with few exceptions, such as HER2muts that our group and others previously identified (23–25), not clinically targetable. As a result, the management of endocrine resistance is still reliant on the ERi+CDK4/6i combination. A major question remaining is whether the HER2-low subtype has relevance in endocrine-resistant disease.

## 2 Intersection of endocrine therapy resistance and HER2-low

**Figure 1:**
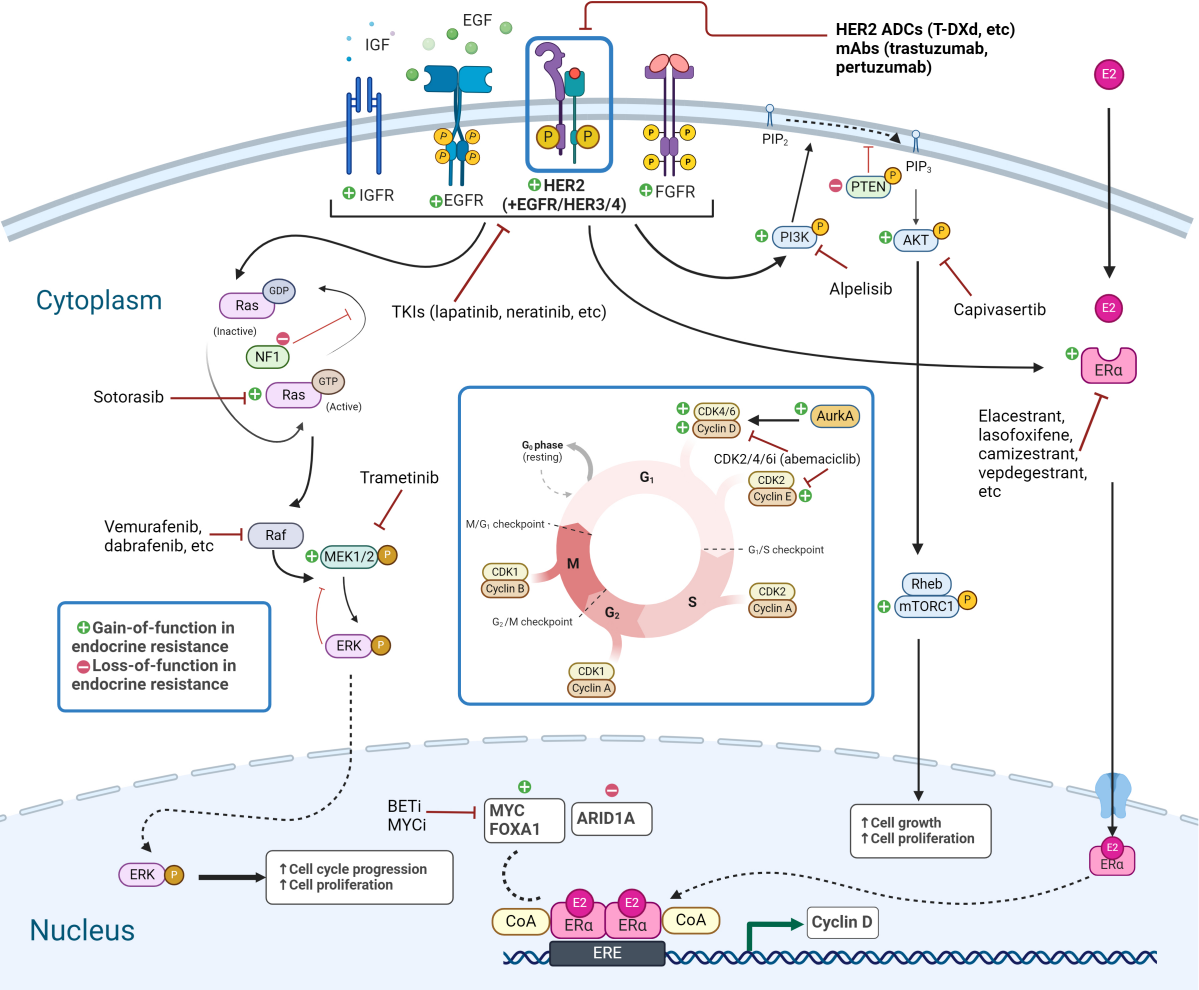
Signaling pathways in endocrine-resistant HER2-low breast cancer. Targetable genetic alterations and pathways in endocrine-resistant HER2-low breast cancer include RTK/MAPK pathway, PI3K/AKT/mTOR pathway, and TFs, along with the ER pathway and transcriptional targets, and alterations related to CDK4/6i resistance. Created with BioRender.com. HER2, human epidermal growth factor receptor-2; TFs, transcription factors; ER, estrogen receptor.

Resistance to endocrine therapies can be caused by a wide variety of molecular alterations, as summarized in **Table 1** and depicted in **Figure 1**. They converge into three classes: 1) ER, 2) TF, and 3) MAPK (14). These include alterations in *ESR1*, *ERBB2*, EGFR, *KRAS*, *FGFR1/2/4*, and ER transcriptional regulators, for instance, *MYC*, *CTCF*, *FOXA1*, and *TBX3*. Simultaneously, genomic and transcriptomic analyses of HER2-low tumors indicate that they harbor several of these same molecular alterations (mutations, amplification/deletion, and fusions) in both the primary (2) and metastatic (26) settings. While the exact frequency remains to be determined, the majority of evidence suggests that a non-insignificant proportion of endocrine-resistant tumors may indeed be HER2-low. We summarize here recent evidence linking endocrine resistance-associated molecular alterations with HER2-low status. To facilitate this, we retrieved tumor-level HER2 immunohistochemistry (IHC) and fluorescence *in situ* hybridization (FISH) data from a comprehensive 2018 study of the genomic landscape of endocrine resistance by Razavi et al. (14) from cBioPortal (27–29) (**Supplementary Table 1**) to identify HER2-low tumors among those bearing validated endocrine resistance-associated genetic alterations (**Supplementary Table 2**). As shown in **Table 1**, a significant proportion of tumors bearing endocrine resistance-associated alterations could be classified as HER2-low (**Supplementary Table 3**), in most cases at a higher frequency (~63%–70%) than the expected proportion of HER2-low tumors (~45%–55%).

**Table 1:** Intersection of endocrine resistance mechanisms and HER2-low.

Alteration	% altered (of n)	%HLBC	Resistance	Relevant clinical trials and/or inhibitors	Refs
*AKT1*Amp/mut	*7% (of 1501)*, 12% (of 41)	63% (66/105)	ER, CDK4/6	CAPItello-291 (AKTi capivasertib), DESTINY Breast-08 (capivasertib)	(14, 52, 53, 111)
*AKT2*Amp	*0.1% (of 1501)*	67% (10/15)	N/A	CAPItello-291 (AKTi capivasertib), DESTINY Breast-08 (capivasertib)	(14, 44, 111)
*ARID1*Amut	*6% (of 1501)*	63% (63/100)	ER	ATRi berzosertib (NCT03718091)	(14, 44, 114, 121)
*AURKA*Amp	27.0% (of 41)	67% (43/64)	ER, CDK4/6	TBCRC041 (AURKAi alisertib)	(53, 122)
*BRAF*mut	0.6% (of 692)	45% (5/11)	ER	EVESOR (BRAFi sorafenib+mTORi everolimus), TAPUR (MEKi cobimetinib+BRAFi vemurafenib), alpelisib (PI3Ki)+binimetinib (MEKi)	(14, 123, 124)
*CCND1*Amp	27.0% (of 41)	70% (240/345)	ER, CDK4/6	N/A	(52, 53)
*CCNE2*Amp	17.0% (of 41)	ND	ER, CDK4/6	CDK2i	(53, 79, 136)
*CTCF*mut	1% (of 692)	58% (18/31)	ER	N/A	(14)
EGFRAmp	2.0% (of 692)	64% (14/22)	ER	Duligotuzumab (EGFR+HER3i), POTENTIATE (CHK1i BBI-355)	(14, 125)
*ERBB2*mut	4%–10%	57% (44/77)	ER, CDK4/6	SUMMIT (HER2i neratinib+HER2i trastuzumab+fulvestrant), DESTINY-Breast04 (HER2i T-DXd)	(14, 23, 24, 56, 96, 100)
*ESR1*mut	18% (of 692), 36% (of 44)	61% (89/147)	ER	EMERALD (ERi elacestrant), SERENA-1 (ERi camizestrant), AMEERA-5 (ERi amcenestrant+palbociclib), ELAINE-1 (ERi lasofoxifene)	(14, 17–20, 33, 104, 105, 126, 127)
*FGFR1*Amp	15% (of 60)17% (12/72)	65% (140/215)	ER, CDK4/6	FOENIX-MBC2 (FGFRi futibatinib), BOLERO-2 (mTORi everolimus), FINESSE (FGFRi lucitanib)	(52, 61–63, 115–117, 120, 128, 129)
*FGFR2*Amp/mut	8.3% (of 60)	57% (24/42)	ER, CDK4/6	FOENIX-MBC2 (FGFR1i futibatinib), BOLERO-2 (mTORi everolimus)	(52, 116, 120, 128)
*FOXA1*mut/Amp	2% (of 692)	67% (51/76)	ER	N/A	(14, 39–41)
IGFRAmp	10% (of 41)	56% (22/39)	ER, CDK4/6	IGF1Ri dalotuzumab	(53, 65, 130)
*KRAS*mut	1% (of 692)	67% (8/12)	ER	KRYSTAL 1 (KRASmuti adagrasib), KRASi sorafenib+mTORi everolimus	(14, 53, 131, 132)
*MAP2K1*mut	0.3% (of 692)	56% (5/9)	ER	MEKi mirdametinib	(14, 133)
*MYC*Amp	9% (of 692)	64% (100/157)	ER	ATRi berzosertib (NCT03718091), CDK9i	(14, 47, 121)
*NF1*mut/del	5% (of 692), *8.1% (17/246)*	56% (52/93)	ER	EAY191-N2 ComboMATCH (fulvestrant+binimetinib MEKi NCT05554354)	(14, 50)
*PIK3CA*mut	*41% (of 1501)*	65% (445/686)	ER	SOLAR-I (PI3Ki alpelisib), CAPItello-291 (AKTi capivasertib, DESTINY Breast-08, NCI-MATCH (PI3Ki copanlisib, alpelisib), mTORi sirolimus	(14, 108–111, 119, 134, 135)
*PTEN*mut/del	*9% (of 1501)*	68% (110/163)	N/A	CAPItello-291 (AKTi capivasertib+fulvestrant), DESTINY Breast-08 (HER2i T-DXd+AKTi capivasertib), copanlisib	(14, 77, 111, 119, 134)
*TBX3*mut	1% (of 692)	63% (54/86)	ER	N/A	(14)

For each endocrine resistance-associated genetic alteration, columns from left to right are the % of endocrine-resistant *(or advanced)* ER+ MBC with the alteration, % of altered tumors in the Razavi et al. (2018) cohort that were HER2-low BC, clinical resistance associated with alteration, relevant clinical trials and/or clinical/pre-clinical inhibitors in ER+ MBC bearing alteration, and references.

HER2, human epidermal growth factor receptor-2; ER+, estrogen receptor positive; MBC, metastatic breast cancer.

### 2.1 ER pathway hyperactivation

Acquired activating hotspot ERmuts are the most well-established mechanism of clinical endocrine resistance—they are dramatically enriched in this setting (~18%–50% of tumors) compared to primary BC (~2%) and primarily confer resistance to AIs (14, 17–20). *In vitro* studies found that ERmuts drive altered (often increased) HER2 downstream signaling (21, 30), thus hinting at potential crosstalk that may be targetable. Some alleles are more active—in the PALOMA-3 study, the Y537S ERmut was associated with resistance to fulvestrant alone and in combination with palbociclib (31, 32). A recent study identified a novel ERmut at F404 in *cis* with activating ERmuts as an acquired mechanism of resistance to fulvestrant in ~4% of tumors (33). ERmuts have been shown to drive an altered ER cistrome through stabilization of the agonist state (34), enabling ER binding to coactivators in the absence of ligand and enhanced binding to estrogen response elements (EREs) in target gene promoters (21), which maintain ER downstream signaling. ERmuts also have a lower affinity for the inhibitor tamoxifen (34). In addition to mutations, *ESR1* overexpression and novel oncofusions (15, 16) can also drive resistance to endocrine therapies.

The recent genomic characterization study of HER2-low metastatic breast cancer (MBC) noted that ERmuts were preferentially enriched in ER+HER2-low tumors over ER+HER2-0 (16% of 370 vs. 11% of 336) (26). In addition, *ESR1* amplification was one of only two copy number variations (CNVs) that were significantly enriched in HER2-low tumors regardless of ER status in this study. A related study in HER2-low tumors found that ER expression levels were significantly higher in this subset (3). Thus, endocrine resistance through ER hyperactivation appears to intersect significantly with HER2-low. In the Razavi cohort, 61% (89/147) of ERmut tumors were HER2-low (14).

### 2.2 Transcription factor alterations

Amplifications and/or hotspot mutations of key transcriptional regulators *ARID1A*, *MYC*, *CTCF*, *FOXA1*, and *TBX3* are candidate mechanisms of resistance in 9% of endocrine-resistant tumors (14).

FOXA1 is a pioneer factor of ER function on chromatin and a known driver of ER+ BC (35–37). FOXA1 and ER co-expressed at high levels in endocrine therapy-resistant MBC (38), and *FOXA1*Amp contributes to endocrine resistance in preclinical models by reprogramming the ER and FOXA1 cistrome and transcriptome (39) and inducing a pro-metastatic secretome (40). *FOXA1*muts were also enriched in MBC and associated with worse responses to AIs (41). Increased *FOXA1* expression levels through promoter mutation create stronger binding for E2F family TFs and promote cellular tolerance to fulvestrant treatment (42).

*ARID1A* is the most frequently mutated subunit of the SWI/SNF chromatin remodeling complex in ER+ BC (43) and an essential luminal lineage driver gene. Loss-of-function *ARID1A*muts were enriched in the post-endocrine metastatic setting and were shown to mediate endocrine resistance *in vitro* by changing the chromatin accessibility of TFs playing a role in luminal differentiation as well as binding of the chromatin remodeling complex SWI/SNF at ER-FOXA1-GATA3 sites (14, 44).

MYC is an ER-regulated gene, whose overexpression confers endocrine resistance by promoting cell cycle progression and altered metabolism/unfolded protein response modulation (45), while knockdown of MYC impairs hormone-independent growth of BC cell lines *in vitro* (46–49).

In HER2-low primary BC, *ARID1A*, *MYC*, *CTCF*, *FOXA1*, and *TBX3* were altered in 15% of tumors, while the frequency in HER2-low MBC remains to be determined (2). *FOXA1* expression was significantly higher in HER2-low primary tumors (3). ARID1A-altered tumors accounted for 4% of HER2-low primary BC (all LumB tumors with a HER2 IHC score of 2+) and 8% of ER+HER2-low MBC (2, 26). MYC copy number gain has been identified in HER2-low tumors (2, 26). Analysis of advanced tumors in cBioPortal (**Table 1**) revealed that of tumors bearing *FOXA1*muts, *MYC*Amp, or *ARID1A*muts, 67% (51/76), 64% (100/157), and 63% (63/100), respectively, can be classified as HER2-low. Among *CTCF*mut and *TBX3*mut tumors, 58% (18/31) and 63% (54/86) were HER2-low.

### 2.3 MAPK/PI3K-AKT pathway hyperactivation

#### 2.3.1 *RAS/RAF/MEK/ERK* pathway

Components of the MAPK signaling cascade *KRAS*, *BRAF*, and MEK1; the negative regular *NF1*; and upstream RTKs (*EGFR*, HER2, *FGFR1/2/4*) are altered in ~13% of endocrine-resistant MBC (14, 50). Several of these are far more prevalent in heavily pre-treated populations by virtue of their ability to confer cross-resistance to not only second-line endocrine therapies but also CDK4/6is.

*KRAS*muts were found in 15.4% of AI-resistant tumors by circulating tumor DNA (ctDNA) analysis, were acquired in endocrine-resistant MBC (14), and conferred resistance to fulvestrant *in vitro* (51, 52). *In vitro*, the *KRAS* G12D activating mutation confers resistance to fulvestrant (53). Of *KRAS*mut endocrine-resistant tumors in the Razavi et al. cohort, 67% (8/12) were HER2-low (**Table 1**) (14).

HER2 (*ERBB2*) and *NF1* were most significantly increased in mutational frequency between pre- and post-hormonal therapy in ER+HER2− MBC (14). *NF1* loss-of-function (LOF) mutations (*NF1*muts) account for 4.6% of endocrine-resistant tumors and drive endocrine resistance *in vitro* through both ER-dependent MAPK pathway-driven expression of Cyclin D1 and ER-independent S-phase entry (50, 54). Although *NF1*-altered tumors in Razavi et al. included several with the HER2-low subtype (**Table 1**, 52/93, 67%), *NF1* loss was strongly associated with HER2-0 status in the HER2-low genomic characterization study (26); thus, this association is of uncertain relevance.

Among the RTK family proteins found altered in endocrine-resistant BC, HER2muts represent a mechanism with unique features. We and others previously identified acquired activating somatic hotspot HER2muts that conferred strong endocrine resistance to all clinically approved ER inhibitors and cross-resistance to the CDK4/6i palbociclib by activating MAPK/PI3K downstream pathways and altering the ER transcriptome (23, 55, 56). Transcriptomic analysis of HER2mut tumors revealed they have higher average HER2 expression than non-HER2mut tumors (57). Similarly, the recent HER2-low study determined that HER2mut tumors were slightly increased in ER+HER2-low tumors (26). We found that 57% (44/77) of HER2mut tumors in the Razavi cohort were HER2-low (**Table 1**). HER2mut tumors were also preferentially enriched in lobular vs. ductal subtypes (58, 59). Lastly, PAM50 analysis of HER2mut tumors appeared to indicate a switch to HER2-E or LumB (23, 60). Thus, ER+HER2mut tumors represent a unique subset of endocrine-resistant tumors that intersect with HER2-low. Mutational landscape analysis of primary HER2-low BC validated HER2muts (8%) (2), while they were also enriched in ER+HER2-low over ER+HER2-0 MBC (26).

##### 2.3.1.1 Other RTKs

*FGFR1/2*Amp and *FGFR2*muts have been identified as a clinical mechanism of resistance to endocrine therapy through ER reprogramming and activation of the MAPK pathway in up to 40% of some smaller cohorts and validated *in vitro* (52, 61–63). *FGFR2*Amp was much more prevalent in HER2-low MBC compared to HER2-0 (26). As MAPK upstream regulators, EGFRAmp has been reported in 1.7% of endocrine-resistant MBC and demonstrated preclinically to confer endocrine (14) and CDK4/6i resistance (64) via activation of ERK and AKT. Additionally, the *IGF1R* pathway has been shown as a mechanism of escape from hormone dependence in BC *in vitro* (65) via crosstalk with the EGFR (66). Significantly, *IGF1R*Amp was one of only two CNVs that were found to be significantly enriched in HER2-low tumors regardless of the ER level (26). cBioPortal analysis revealed that 64% of MBC with *FGFR1/2* gene alterations (164/257) and EGFRAmp (14/22) were HER2-low (**Table 1**), along with 56% (22/39) of *IGF1R*Amp.

#### 2.3.2 *PI3K/AKT* pathway

The PI3K pathway components *PIK3CA*, *AKT1/2*, and *PTEN* are highly mutated in ER+ BC (67, 68). The role of *PIK3CA*muts in endocrine resistance is unclear. Clinically, based on the PALOMA-3 study, there was no significant difference in progression-free survival (PFS) between *PIK3CA*mut and *PIK3CA*wt tumors (4), and there was no significant enrichment of *PIK3CA*muts in endocrine-resistant MBC (14). However, *PIK3CA*muts can confer endocrine resistance *in vitro* (69). Genomic characterization of HER2-low tumors indicated that the frequency of *PIK3CA*muts was similar in ER+ tumors regardless of HER2-low or HER2-0 status (26). Of ER+ MBC tumors with *PIK3CA*muts, 65% are HER2-low (14) (**Table 1**), but HER2-low MBC has a lower frequency of *PIK3CA*muts overall (26).

AKT is a downstream effector of PI3K, whose overexpression confers endocrine and CDK4/6i resistance *in vitro* (52, 53). Clinically, *AKT1* alterations account for 7% of ER+HER2− MBC (14) and were enriched compared with primary disease (50). Some studies suggested that activated AKT (increased pAKT) levels negatively correlated with the efficacy of endocrine therapy (70) and were associated with worse outcomes among endocrine-treated BC patients (71, 72). *AKT1*muts were highly enriched in HER2-low MBC when compared with HER2-0 (26).

mTOR activation is highly correlated with a lack of response to ER+CDK4/6i in clinical studies (73) and increased migration and stemness (74). While mTORmuts have not been validated as a mechanism of endocrine resistance *in vitro*, they have been found to be acquired in ER+CDK4/6i-resistant tumors (60). In a genomic study, *MTOR* was the top significant gene that was mutated preferentially in HER2-low vs. HER2-0 MBC (26).

*PTEN* is a negative regulator of the PI3K pathway, and its loss is suggested to contribute to clinical endocrine resistance by activating PI3K signaling (75, 76). *In vitro*, reduced PTEN levels resulted in endocrine resistance (77), while *PTEN* loss was enriched in HER2-low vs. HER2-0 MBC (26).

Analysis of advanced tumors in the Razavi cohort (**Table 1**) revealed that 63% of *AKT1*-altered (66/105), 67% of *AKT2*Amp (10/15), 65% of *PIK3CA*mut (445/686), and 67% of PTEN-altered tumors can be classified as HER2-low.

### 2.4 Endocrine and CDK4/6i combination therapy resistance

CDK4/6is block the cell cycle transition from the G1 phase to the S phase, leading to cell cycle arrest in ER+ BC (78). However, resistance to CDK4/6is occurs in virtually all patients, leading to high mortality. In ER+ BC, CDK4/6is are usually utilized in combination with ERis—studies of acquired clinical resistance to the combination found that acquired alterations in *RB1*, *CCND1*, *CDK4/6*, *CCNE1/2*, and *AURKA* were enriched in resistant tumors (53, 79). Additionally, some established mechanisms of endocrine resistance, specifically those in the “MAPK” class such as *ERBB2*muts and *IGF1R*Amp (80), *AKT1*Amp, and *AKT*1muts, confer cross-resistance to CDK4/6is. Key recent findings indicate that the HER2-low subtype is independently associated with worse response to ERi+CDK4/6i, while the specific molecular alterations enriched have not been determined. The recent genomic characterization of HER2-low MBC indicated that *CCND1* and *CDK4* were more frequently amplified in HER2-low over HER2-0 as a potential mechanism of CDK4/6 resistance (26). Thus, ER+HER2-low tumors represent a sizeable fraction of ER+ tumors that are particularly intractable to ER+CDK4/6is in the clinic and for which therapeutic options are desperately needed.

### 2.5 Subtype switching in endocrine-resistant tumors

The PAM50 intrinsic subtype (IS) was the strongest prognostic factor predicting PFS and OS (LumA > LumB > HER2-E/basal) in a retrospective analysis of ER+ MBC (81), while another analysis of clinical trials in ER+ MBC showed that the HER2-E subtype correlated with response to HER2i and ribociclib, and the basal subtype correlated with response to chemotherapy (82). Endocrine resistance is also linked with enhanced phenotypic plasticity (83), which enables IS switching through downregulation of luminal markers and upregulation of basal markers (84), and transition to a non-luminal subtype (basal or HER2-E). Switching between HER2+ and HER2− is relatively common (~36%) in studies of primary vs. metastatic tumors from the same patient (85), along with IS switching—there is enrichment of HER2-E and decrease of LumA in MBC (86). Gene expression changes and subsequent IS switches were more frequent in LumA MBC than in the other molecular subtypes (87). For example, 40.4% of LumA switched to LumB, and 10%–15% of LumA/B tumors switched to HER2-E in a retrospective study (82), while 90% of LumA tumors switched to LumB or HER2-E in the AURORA study of MBC (88). Another study of paired primary and relapse (endocrine-refractory) tumors found that 19/70 (27.1%) underwent the PAM50 switch, with the HER2-E subtype enriched in the resistant setting (89).

Additionally, there is a switching of HER2-low status in progressive disease—a retrospective study of LumB BC patients showed that there was extensive switching between HER2-low and HER2-0 status at a rate of 34% or 25% in either direction (90). Endocrine-resistant ER+ MBC often undergoes switches between HER2-0 and HER2-low status (90).

While the exact percentage remains to be determined due to a lack of systematic large-scale clinical transcriptomic studies, there is limited evidence that tumors with acquired endocrine resistance-associated genetic alterations undergo subtype switches. For example, a prior study from our group showed that an ER+ MBC tumor that acquired a HER2mut switched from ER+HER2-0 to ER+HER2-low (23), while *HER2*mut tumors are clinically associated with HER2-E (88). Although transcriptomic characterization of primary tumors linked the HER2-low subtype most closely with a LumA signature (3), these may differ in the endocrine resistance context. Along these lines, a recent small study in ER+ MBC showed that most ERmut, *FGFR1*Amp, *NF1*loss, *AKT1*mut, and *CCND1*Amp tumors were predominantly LumB, while the HER2mut and *BRAF*mut tumors in this cohort were HER2-E, rather than LumA (60). *FGFR*Amp, EGFRAmp, and *CCND1*Amp were also enriched in HER2-E primary tumors (91). Additionally, all the ERmut tumors in another study of primary HER2-low tumors were classified as the LumB subtype and were characterized by a HER2 IHC score of 2+ (2). While *PIK3CA*muts were not associated with endocrine resistance, they were preferentially enriched in MBC undergoing a subtype switch to HER2-E in AURORA (88).

Alterations that lead to subtype switch to basal may be less likely to have relevance to HER2-low signaling, although several of these are significantly associated with HER2-low in the MBC setting (26). For example, ERmut (92) and *ARID1A*mut (44) tumors demonstrate upregulation of basal genes*. GATA3*, while not enriched in endocrine resistance, is an important marker of ER dependence and luminal identity (93)—*GATA3*muts were preferentially enriched in ER+HER2-low tumors over ER+HER2-0 (16% of 370 vs. 9% of 336) (26) while also present in ~18% (18/99) of HER2-low primary BC (2).

Importantly, HER2i was shown to be able to convert HER2-E tumors to less aggressive LumA in HER2+ MBC (94), which may enable re-sensitization to endocrine therapy. Thus, subtype switching in endocrine-resistant HER2-low tumors, particularly to HER2-E, may be able to inform treatment.

## 3 Potential therapeutic strategies for endocrine-resistant HER2-low tumors

Historical evidence confirmed that HER2 expression or amplification level correlates strongly with response to and benefit from trastuzumab (95), lending credence to HER2 as a biomarker. In contrast, the DESTINY-Breast04 (96) and DAISY (97) trials demonstrated the surprising benefits of treating HER2-low MBC with the HER2 antibody–drug conjugate (ADC) trastuzumab deruxtecan (T-DXd). Deruxtecan is unique in its ability to kill tumor cells nearby, demonstrating a potent bystander effect due to its membrane-permeable payload (98). Patients with refractory ER+ BC who received T-DXd had significantly longer progression-free and overall survival than those who received chemotherapy (96). Surprisingly, this efficacy was independent of HER2 status. Based on this trial, T-DXd was approved in August 2022, demonstrating an improvement in the clinic for those diagnosed with ER+HER2-low MBC.

Endocrine resistance-associated genetic alterations differ widely in their profiles of response to targeted therapies (**Table 1**). HER2mut tumors were cross-resistant to the CDK4/6i palbociclib (23) and variably sensitive to HER2 tyrosine kinase inhibitors (TKIs) (55), while the irreversible TKI neratinib resensitized HER2muts to fulvestrant *in vitro*. The combination of neratinib with trastuzumab and fulvestrant was efficacious against HER2mut ER+ BC in the SUMMIT basket trial (23, 25, 99), after progression on CDK4/6is (100), and the fulvestrant+neratinib combination in ER+HER2mut was further supported by the mutHER trial (101). Finally, the DESTINY-Lung02 trial also indicated that T-DXd had surprising activity in HER2mut tumors (102), potentially due to increased mutant receptor internalization (103).

The standard of care treatment for ER+HER2− BC remains the combination of ERi+CDK4/6i in the advanced setting. Patients who developed resistance to therapy by acquiring ERmuts can now benefit from novel oral SERDS, such as elacestrant and camizestrant (104)—the former was FDA-approved specifically for ER+HER2− ERmut MBC in 2023. Newer ER-targeting agents also have been found to have activity against ERmuts—the phase II ELAINE 1 trial demonstrated encouraging antitumor activity of lasofoxifene, a novel SERM/SERD hybrid, in patients with ERmut endocrine-resistant MBC following progression on AI+CDK4/6i (105). Newer ER proteolysis-targeting chimeras (PROTACs) like vepdegestrant also appear promising (106). Aside from newer SERDs, some TF-driven endocrine resistance may continue to respond to other ER-targeting agents. For example, *FOXA1*muts showed sensitivity to tamoxifen or fulvestrant *in vitro* (41). Although ERmuts are associated with poor response to CDK4/6is, abemaciclib, which is unique compared to other CDK4/6is, may be of use, particularly with the deployment of a recently developed multigene panel that could predict resistance to abemaciclib in ERmut tumors (107).

Clinically, *PIK3CA*muts are targetable through the PI3K inhibitor, alpelisib (108). In ER+HER2− MBC, patients with *PIK3CA*mut tumors had significantly longer PFS with alpelisib+fulvestrant than fulvestrant alone. The FDA approved alpelisib+ fulvestrant for *PIK3CA*mut ER+HER2- MBC patients based on overall survival results from the SOLAR-1 trial (109). In the case of endocrine therapy resistance associated with reduced PTEN levels, adding mTOR (110), AKT, or MEK inhibitors to endocrine therapy has been suggested as a promising strategy *in vitro* (77). PI3K pathway alterations are additionally targetable through the newest approved pan-AKT inhibitor capivasertib (111, 112), which is currently being evaluated in combination with T-DXd for HER2-low BC in the phase III DESTINY-Breast08 trial (113).

Other mechanisms lack clinically approved or defined targeted therapeutic strategies at the moment. These include *ARID1A* loss, which sensitized ER+ cells to BET inhibition *in vitro* (114). Targeting FGFR with an irreversible pan-FGFR inhibitor, FIIN-3 reversed the activation of FGFR and resensitized cells expressing FGFR2 to fulvestrant (52). *FGFR1* overexpressing ER+ BC cells were highly sensitive to the mTOR inhibitor everolimus (115). The combination of fulvestrant with either everolimus or with the FGFR TKi lucitanib overcame endocrine resistance *in vitro* (115). Futibatinib, an irreversible FGFR inhibitor, significantly inhibited the growth of PDX models bearing FGFR2 alterations (116). The FOENIX-MBC2 ongoing clinical trial demonstrated the efficacy of futibatinib in patients with locally advanced/metastatic BC harboring *FGFR1/2* amplifications who have experienced disease progression after prior therapy for advanced/metastatic disease (117). Other pan-FGFR inhibitors are in clinical trials for FGFR-altered tumors (118).

In most of the abovementioned endocrine resistance-associated altered tumors, classification as HER2-low opens up additional therapeutic strategies that could be advantageous, particularly in cases where dependence on HER2 is borne out by PAM50 subtyping and/or subtype enrichment. Several key clinical trials with relevance to specific genetic alterations are included in **Table 1**. These include the DESTINY-Breast04 (96) and DESTINY-Breast08 (119) trials, which are in HER2-low MBC and are likely to include endocrine-resistant tumors, as well as others in which specified genetic alterations were inclusion criteria, or in which retrospective subgroup analysis has indicated the significant benefit of the intervention for the altered tumors (22, 100, 105, 109, 111, 117, 120–137).

## 4 Discussion (perspective)

Overall, the available clinical data indicate that a significant subset of endocrine-resistant tumors may be characterized as HER2-low in opposition to HER2-negative. With the advent of newer HER2-targeting therapies such as T-DXd, the time is ripe to consider whether combination regimens that include HER2-targeting ADCs should be explored in combination with endocrine agents, especially novel oral SERDs, as next-line therapies for patients failing ER and CDK4/6 inhibition. There are two reasons that this approach should be considered: 1) the vast diversity in mechanisms of acquired endocrine and CDK4/6 clinical resistance mechanisms makes it challenging to target tumors effectively, and 2) as the two major molecularly targeted pathways in breast cancer, there is a continuous pipeline of newer ER and HER2 inhibitors that may be potentially viable for endocrine-resistant HER2-low tumors. The major questions that remain to be addressed include 1) what proportion of endocrine-resistant tumors may be classified as HER2-low, 2) which specific genetic alterations are enriched in this context that may be targetable in addition, and 3) whether endocrine-resistant HER2-low tumors tend to be preferentially lobular vs. ductal. The landscape of lobular carcinoma is beginning to be understood as a distinct entity (138), and certain alterations such as HER2muts are enriched in lobular as well as among HER2-low. Thus, our ability to exploit HER2-low status in endocrine resistance will depend on a more comprehensive understanding of the disease context for ER+HER2-low tumors.

## Data Availability

Publicly available datasets were analyzed in this study. This data can be found here: https://www.cbioportal.org/study/summary?id=breast_msk_2018.
